# Isolation and characterization of an osmotic stress and ABA induced histone deacetylase in *Arachis hygogaea*

**DOI:** 10.3389/fpls.2015.00512

**Published:** 2015-07-13

**Authors:** Liang-Chen Su, Bin Deng, Shuai Liu, Li-Mei Li, Bo Hu, Yu-Ting Zhong, Ling Li

**Affiliations:** Guangdong Provincial Key Laboratory of Biotechnology for Plant Development, School of Life Sciences, South China Normal UniversityGuangzhou, China

**Keywords:** epigenetics, ABA, osmotic stress, acetylation, HDAC, RNA-seq, TSA

## Abstract

Histone acetylation, which together with histone methylation regulates gene activity in response to stress, is an important epigenetic modification. There is an increasing research focus on histone acetylation in crops, but there is no information to date in peanut (*Arachis hypogaea*). We showed that osmotic stress and ABA affect the acetylation of histone H3 loci in peanut seedlings by immunoblotting experiments. Using RNA-seq data for peanut, we found a RPD3/HDA1-like superfamily histone deacetylase (HDAC), termed AhHDA1, whose gene is up-regulated by PEG-induced water limitation and ABA signaling. We isolated and characterized *AhHDA1* from *A. hypogaea*, showing that AhHDA1 is very similar to an Arabidopsis HDAC (AtHDA6) and, in recombinant form, possesses HDAC activity. To understand whether and how osmotic stress and ABA mediate the peanut stress response by epigenetics, the expression of *AhHDA1* and stress-responsive genes following treatment with PEG, ABA, and the specific HDAC inhibitor trichostatin A (TSA) were analyzed. *AhHDA1* transcript levels were enhanced by all three treatments, as was expression of peanut transcription factor genes, indicating that AhHDA1 might be involved in the epigenetic regulation of stress resistance genes that comprise the responses to osmotic stress and ABA.

## Introduction

Plants respond to various abiotic stresses by altering the expression of many genes. Drought is one of the most significant of such abiotic stresses because it limits cell growth and development; consequently, plants have developed diverse strategies to cope with limited water availability (Jung et al., 2005). One such strategy is epigenetic modification of chromatin structure through post-translational modification of histones, for example by acetylation and ubiquitination of lysine residues, methylation of arginine, and phosphorylation of serine or threonine (Henderson and Jacobsen, 2007; Kim et al., 2010). This regulates the expression of genes within the modified chromatin, thereby affecting plant growth and development (Lopez-Gonzalez et al., 2014; Zhang et al., 2014).

Histone acetylation is controlled by histone acetyltransferases (HATs) and histone deacetylases (HDAs or HDACs). In general, HATs transfer acetyl groups to core histone tails, thereby promoting transcription of target genes, whereas HDACs remove acetyl groups from the Lys residues of histone tails, resulting in the repression of gene transcription (Kurdistani and Grunstein, 2003). Plant HDACs are classified into three distinct families, namely RPD3/HDA1-like HDAs, SIR2-like HDAs, and HD2 proteins, based on sequence similarity, substrate specificity, and cofactor requirement (Pandey et al., 2002; Loidl, 2004; Fong et al., 2006; Zhong et al., 2013). *Arabidopsis thaliana* has 12 RPD3/HDA1 subfamily genes (*HDA2, HDA5, HDA6, HDA7, HDA8, HDA9, HDA10, HDA14, HDA15, HDA17, HDA18*, and *HDA19*) among 18 putative HDAC family genes (Ma et al., 2013). Of these, *HDA6* has been reported to participate in jasmonic acid-mediated plant defense responses and to be involved in transgene silencing and the regulation of rRNA transcription (Murfett et al., 2001; Devoto et al., 2002; Tanaka et al., 2008); *HDA19* is involved in jasmonic acid and ethylene signaling during the response to pathogens, and redundantly with *HDA6* regulates the repression of embryonic properties during germination (Zhou et al., 2005; Tanaka et al., 2008); both up-regulation and down-regulation of *HDA7* and *HDA18* in Arabidopsis cause growth delays at different developmental stages (Cigliano et al., 2013; Liu et al., 2013); *HDA9*, which acts to oppose the effect of its homologs *HDA6* and *HDA19*, is a negative regulator of germination in seedlings (van Zanten et al., 2014). Thus, these HDAs respond to environmental stress or participate in plant development.

HDAs function on various histone loci within chromatin and these can be detected by Western blot, chromatin immunoprecipitation (ChIP) assays or immunocytochemistry. Research in plants has focused on modifications of histones H3 and H4, which are involved in cell development, flowering, transposon repression and abiotic stress response (Zhao et al., 2014). In Arabidopsis, there is region-specific enrichment of H3K23ac and H3K27ac in the coding regions of the drought-responsive genes *RD29B, RD20*, and *RAP2.4*, while enrichment of H3K4me3 and H3K9ac correlates with *RD29A, RD29B, RD20*, and *RAP2.4* gene activation in response to drought stress (Kim et al., 2008). DREB1 (dehydration responsive element binding 1) proteins, have been shown to play an important role in the response of plants to low-temperature stress (Liu et al., 1998). During cold stress in rice, histone H3K9 acetylation is increased throughout the 800 bp region of *OsDREB1b*, whereas H3K14 and K27 acetylation is biased more toward the core promoter and upstream region, respectively (Roy et al., 2014). Immunoblotting analysis shows that H3K9ac, H3K18ac, H3K27ac, and H4K5ac levels increase with the expression of HATs in response to drought treatment in rice leaves (Fang et al., 2014). In maize, H3K9ac, H4K5ac, and H4ac levels in the *ZmICE1* and *ZmCOR413* promoter and coding regions increase with *ZmDREB1* up-regulation on cold treatment (Hu et al., 2011). Thus, modifications in histone acetylation patterns in plants during stress treatment are associated with the expression of stress response genes.

Drought is one of the most growth-limiting factors for crops. In our previous research on the molecular consequences of environmental stress and abscisic acid (ABA) action in peanut (*Arachis hypogaea*), an economically important oil- and protein-rich crop plant, we analyzed the role of drought-related genes under conditions of water limitation. AhNCED1 (9-cis-epoxycarotenoid dioxygenase) protein catalyzes the rate-limiting step in the ABA biosynthetic pathway in peanut, and its expression is up-regulated by dehydration and ABA; furthermore, heterologous expression of *AhNCED1* increases drought resistance in Arabidopsis (Wan and Li, 2005, 2006). In addition, we also found that *AhAREB1*, a gene which encodes a transcription factor (TF), was induced by ABA or drought (Li et al., 2013). Genes encoding stress-combative dehydrins, i.e., *AhDHNs*, were also upregulated by ABA and PEG (which imposes osmotic stress) in peanut leaves (Su et al., 2012). RNA-seq results show that other TF-like genes (*MYB92*-like and *WRKY33*-like) participate in the early stages of the peanut response to ABA and osmotic stress (Li et al., 2014). However, whether any of these genes are involved in epigenetic regulation, specifically with respect to the osmotic stress response, is still unknown and there are very few reports of the relationship between osmotic stress, ABA signals and plant deacetylation in crops.

In this paper, histone acetylation status in peanut was found to be modified as part of the response to both ABA and PEG treatment. By reference to an RNA-seq database for peanut, we discovered a histone deacetylase 6-like gene that was up-regulated by water deficit and ABA (Li et al., 2014), a result we confirmed by quantitative real-time PCR (qRT-PCR). This histone deacetylase sequence, termed *AhHDA1*, was isolated and its expression was analyzed to determine transcripts abundance in different tissues of peanut. The expression of *AhHDA1* was compared to that of various drought resistance genes during osmotic stress and ABA treatment to attempt to understand the role of AhHDA1 under these conditions.

## Materials and methods

### Peanut plants and growth conditions

Seeds of peanut (*Arachis hypogaea* L. cv Yueyou 7) (Fang et al., 2007) were sown in pots with a potting mixture of vermiculite, perlite and soil (1: 1: 1), and grown in a illumination incubator with 16 h of light from fluorescent and incandescent lamps (200 μmol m^−2^s^−1^) at 26°C followed by 8 h of darkness at 22°C. Plants were watered with half-strength Murashige and Skoog nutrient solution every other day (Wan and Li, 2005).

### Abiotic stress and hormone treatments of peanut plants

Four-leaf stage peanut seedlings were treated with PEG6000 (Roche) to simulate osmotic stress conditions, and ABA (Roche) and trichostatin A (TSA, Roche) were also applied exogenously for other treatments. After water had been removed by filter paper, the seedlings were harvested, rinsed with deionized water, and placed in beakers containing different solutions of PEG, ABA, or TSA in deionized water. The seedlings were transferred to an illumination incubator (26°C, 60% moisture) under continuous light. PEG and ABA were applied at a concentration of 20% (w/v) and 100 μM, respectively (Wan et al., 2011). TSA was applied at a concentration of 1 μM. Control plants were planted in soil but not treated. Mock plants were placed in an equivalent volume of deionized water as experimental plants instead of ABA and TSA solutions. Control group and treated groups were also used for qRT-PCR and immunoblotting (see below for details). Peanut leaf samples (100 mg) were taken at 0, 1, 2, 5, and 8 h and were maintained at −70°C until further use.

### Protein gel electrophoresis and immunoblotting

Peanut leaves (800 mg) were ground to a powder in liquid nitrogen and mixed to homogeneity in 1 ml ice-cold extraction buffer 1 (10 mM potassium phosphate, pH 7.0, 0.1 M NaCl, 10 mM beta-mercaptoethanol, 1 M hexylene glycol) with protease inhibitor (Roche, catalog No. 06538304001). The extract was centrifuged at 13,000 rpm for 10 min at 4°C and the supernatant was discarded. The pellet was resuspended gently in 0.5 ml pre-cooled buffer 2 (10 mM potassium phosphate, pH 7.0, 0.1 M NaCl, 10 mM beta-mercaptoethanol, 1 M hexylene glycol, 10 mM MgCl_2_, 0.5% Triton X-100) with protease inhibitor, centrifuged at 13,000 rpm for 10 min at 4°C and the supernatant was discarded. The buffer 2 step was repeated until the supernatant after centrifugation was light green. Then the pellet was resuspended gently in 1 ml pre-cooled buffer 3 (10 mM potassium phosphate, pH 7.0, 0.1 M NaCl, 10 mM beta-mercaptoethanol) with protease inhibitor, centrifuged at 13,000 rpm for 10 min at 4°C and the supernatant was discarded. The nuclear pellet was resuspended gently in 0.5 ml pre-cooled sonication buffer (10 mM potassium phosphate, pH 7.0, 0.1 M NaCl, 10 mM EDTA pH 8.0, 0.5% sarkosyl). The resuspended mixture was sonicated for 5 min on ice and sonicated samples were centrifuged at 13,000 rpm for 5 min at 4°C. The supernatant was transfer into a new tube and stored at −70°C.

The nuclear extract was suspended in 5 × SDS PAGE loading buffer (0.25 M Tris-HCl, pH 6.8, 10% SDS, 50% glycerol, and 5% 2-mercaptoethanol). The concentration of protein samples was determined using a Bio-Rad protein assay kit (Bio-Rad, Hercules, CA, USA), loaded and run on 15% polyacrylamide gels, then gels were blotted onto a 0.22 μm PVDF membrane. The membrane was blocked in Tris-buffered saline with 0.1% Tween 20 (TBST, pH 7.6) containing 5% dry milk overnight and then incubated with 0.01–0.05 mg/mL of anti-histone H3 (Abcam, catalog no. ab1791), anti-acetyl-histone H3 (Abcam, catalog no. ab47915), anti-acetyl-histone H3K9 (Millipore, catalog no. 07-352) and anti-acetyl-histone H3K14 (Millipore, catalog no. 07-353) for 2 h at room temperature. After washing, the primary antibody was detected with secondary goat anti-rabbit alkaline phosphatase-coupled antibody (Millipore, catalog no. AP307A) at room temperature for 45 min. Visualization was achieved using the ECL system (Millipore, catalog no. 345818).

### Isolation and sequence analysis of *AhHDA1* from *Arachis hypogaea* L.

First-strand cDNA was synthesized by reverse transcription (RT) of 1 μg of total RNA from peanut leaves, either untreated or treated for 5 h with 20% PEG 6000, using 200 units Superscript III Reverse Transcriptase (Invitrogen, catalog No. 18080) and 500 ng oligo-dT primer. The cDNA was used as the template for PCR using specific primers (ORF1-F: AAGTTGAAAACCCCACACCT; ORF1-R: CACCAAGCAGACTAAAGCAAAA) for the amplification of *AhHDA1*. These primers were designed to amplify the full length sequence of the *AhHDA1* ORF. RT conditions were: 70°C for 10 min, followed by 42°C for 1 h, followed by 15 min at 70°C. PCR amplification was performed as follows: 94°C for 5 min, then 35 cycles of 94°C for 30 s, 55°C for 45 s and 72°C for 1 min, then finally 72°C for 10 min.

PCR fragments were gel purified with an Agarose Gel DNA Purification Kit (TaKaRa, catalog no. DV805A) and were ligated into the pMD 19-T Vector (TaKaRa, catalog no. 6013). Plasmids were isolated and were sequenced from both strands. Sequence analysis was performed using EditSeq software (Lasergene7.0). Computer analysis of the DNA and amino acid sequences was carried out using the BLAST program at the National Center for Biotechnology Information Services (http://www.Ncbi.Nlm.Nih.gov/BLAST). For phylogenetic analysis, we used neighbor-joining (NJ) methods implemented using the full alignment program in DNAMAN software (Wan and Li, 2005). 3D comparative protein structure models of peanut AhHDA1 were generated with the automatic modeling mode of SWISS-MODEL implemented on the SWISS-MODEL Workspace website (http://swissmodel.expasy.org/) (Schwede et al., 2003; Arnold et al., 2006).

### Quantitative real-time PCR (qRT-PCR)

RNA extraction was carried out as described by Wan and Li (2005). Three biological replicate RNA samples of each time point and treatment were used for downstream applications. First-strand cDNAs, obtained using the Superscript III reverse transcriptase kit with 0.3 nmol random 15-mers for reverse transcription of 1 μg RNA, were used as templates for qRT-PCR. Aliquots of 1 μl cDNA were then used for each RT-qPCR reaction. Absolute QPCR SYBR Green ROX Mix (ABgene, catalog no. AB-4105) was used according to the manufacturer's instructions for quantification with the ABI PRISM 7300 Sequence Detection System (Applied Biosystems, UK). A melting curve confirmed single product amplification. Analysis of the raw data and calculation of the efficiency (E) for every single well was done using the software PCR Miner (Zhao and Fernald, 2005). Relative expression for each well was calculated as (1 + E) − CT (Muller et al., 2002). Expression data for *A. hypogaea* L. was normalized using the geometric mean (geomean) of the validated housekeeping genes, *ACTIN* and *ADH3* (Chi et al., 2012; Reddy et al., 2013): the primers *ACT11*-F and *ACT11*-R, specific to the peanut *ACTIN* gene (GenBank accession no. GO339334), were used to amplify a fragment of 108 bp, and the primers *ADH3*-F and *ADH3*-R, specific to the peanut *ADH3* gene (GenBank accession no. EG529529), were used to amplify a fragment of 143 bp. The mean values shown (±SE) were calculated from three biological replicates. Primers are listed in Table S1.

### Production of recombinant AhHDA1 in *E. coli*

The PCR product of *AhHDA1* was cloned into an *E. coli* expression vector, pPROEX HT (Invitrogen, catalog No. 10711-018) (Pompon et al., 1996). The resulting plasmid was transformed into *E. coli* strain BL21 (Figure S2). Transformants were grown in LB medium (10 g/L Tryptone, 5 g/L yeast extract, 10 g/L sodium chloride, 50 g/mL ampicillin) at 37°C for 8–10 h. Once the OD600 reached 0.7, 0.1 mM IPTG was added to the LB medium. Then the bacterial suspension was placed in a shaking incubator at 16°C for 20 h. To prepare total proteins, *E. coli* cells were collected and suspended in 0.1 mol/L potassium phosphate buffer (pH 7.6). The lysates of the bacterial cells were centrifuged (at 4°C, 10,000 rpm, 10 min), and the supernatants were subjected to Ni-NTA HisTrap FF crude column chromatography for purification of the recombinant protein. The purified protein was dissolved in phosphate-buffered saline (pH 7.6) to a final concentration of 0.8 mg/mL. The purity of the recombinant AhHDA1 protein was analyzed using SDS-PAGE.

### HDAC enzyme activity assay (colorimetric detection)

This two-step procedure was performed in a microtiter plate using an HDAC Assay Kit (Millipore, catalog no. 17-374). Each well-contained 10 μl 2X HDAC assay buffer, or 2X HDAC assay buffer containing 4 μM trichostatin A, to which 20 μl test protein sample, or 20 μl HeLa nuclear extract (positive control; supplied with kit) or 20 μl water (negative control) were added; the plate was then equilibrated at the assay temperature (37°C). After adding 10 μl of the 4 mM HDAC assay substrate and mixing thoroughly, the microtiter plate was incubated at 37°C for 60 min. Then 20 μl of the diluted activator solution was added to each well, mixed thoroughly and the microtiter plate was incubated at room temperature for 15 min. The absorbance was read in a plate reader at 405 nm.

## Results

### PEG and ABA mediate alterations of H3K9 and H3K14 acetylation status in *Arachis hypogaea* L. leaves

**Figure 1:**
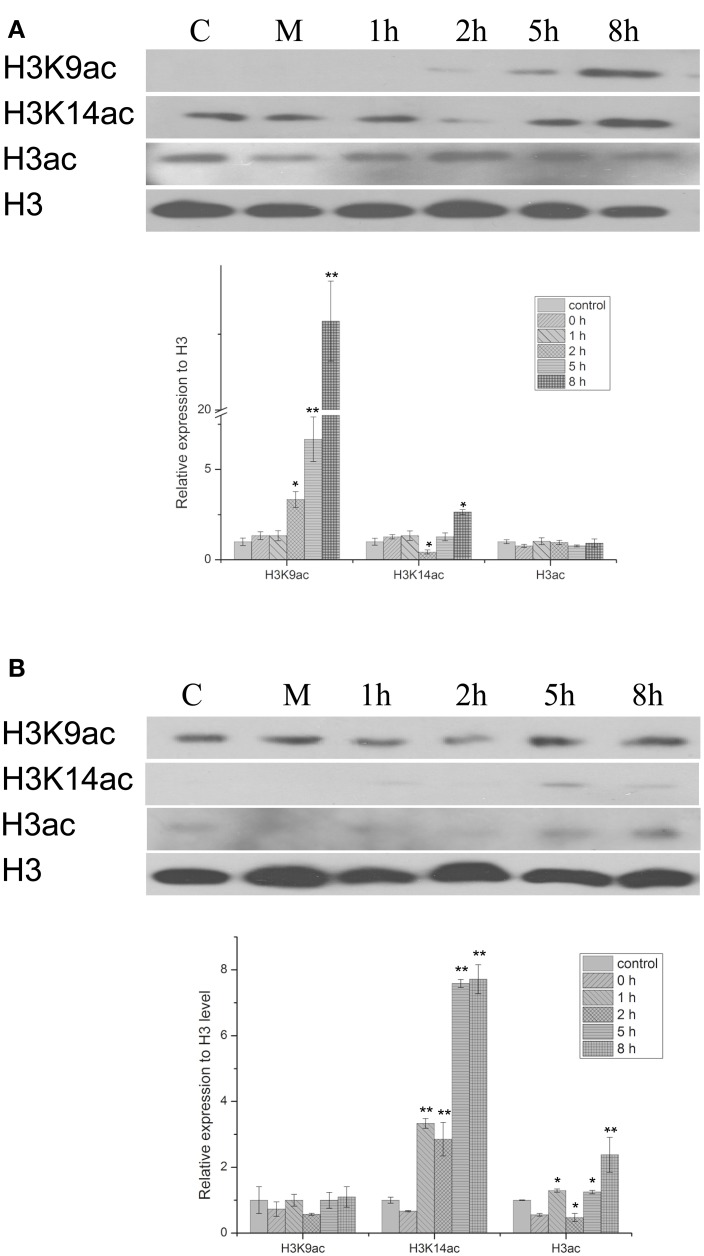
**Western blot showing the effect of PEG and HBA on histone H3 acetylation status in nuclear proteins from peanut leaves**. **(A)** H3 acetylation status in peanut leaves treated with 20% (w/v) PEG. **(B)** H3 acetylation status in peanut leaves treated with 100 μM ABC. C, control group; M, Mock plants were placed in an equivalent volume of deionized water as experimental plants; 1–8 h, time point after treatment. The experiments have been carried out at least three times. Each graph displays the mean and SD of three independent experiments. ^*/**^, different from control as revealed by *t*-test, *p* < 0.05/0.01.

The histone acetylation status of chromatin was investigated in peanut leaves subjected to PEG-induced osmotic stress or to treatment with the stress-protective hormone ABA. Immunoblotting experiments showed that the H3K9ac level increased with 20% PEG treatment, while the H3K14ac level increased with 100 μM ABA treatment (Figure 1). H3K9ac levels following PEG treatment began to increase from 2 h and continued to increase through to the 8 h time point; thus, after 5 h H3K9ac levels showed a significant increase to 7 times that of the control group, and at 8 h had increased further to 23 times control levels. Treatment with PEG produced only a marginal increase in H3K14ac levels at 8 h, but ABA treatment significantly increased the amount of H3K14ac by 5 h to 8 times that of the control group. The results indicate that both PEG and ABA can mediate changes in acetylation at different histone H3 loci; these different patterns of modification suggest that the two treatments result in different gene activation profiles in peanut leaves. At the same time, it has also been proved that the acetylation of H3K9, H3K14, and H3 were increased with 1 μM TSA treatment from 1 to 8 h (Figure S4).

### Isolation and characterization of the peanut *AhHDA1* gene

From the above results, it is clear that osmotic stress and ABA affect the acetylation of histone H3. We therefore screened an RNA-seq database which identifies genes that are differentially expressed following PEG and ABA treatment of peanut (http://www.ncbi.nlm.nih.gov/bioproject/243319) and found a full length ORF of a sequence (*comp66763_c0*) similar to the Arabidopsis *HDA6* gene. According to the RNA-seq data, this gene, named *AhHDA1* (GenBank accession No. KC690279), is inducible by PEG and ABA treatment in peanut leaves from four-leaf seedlings.

**Figure 2:**
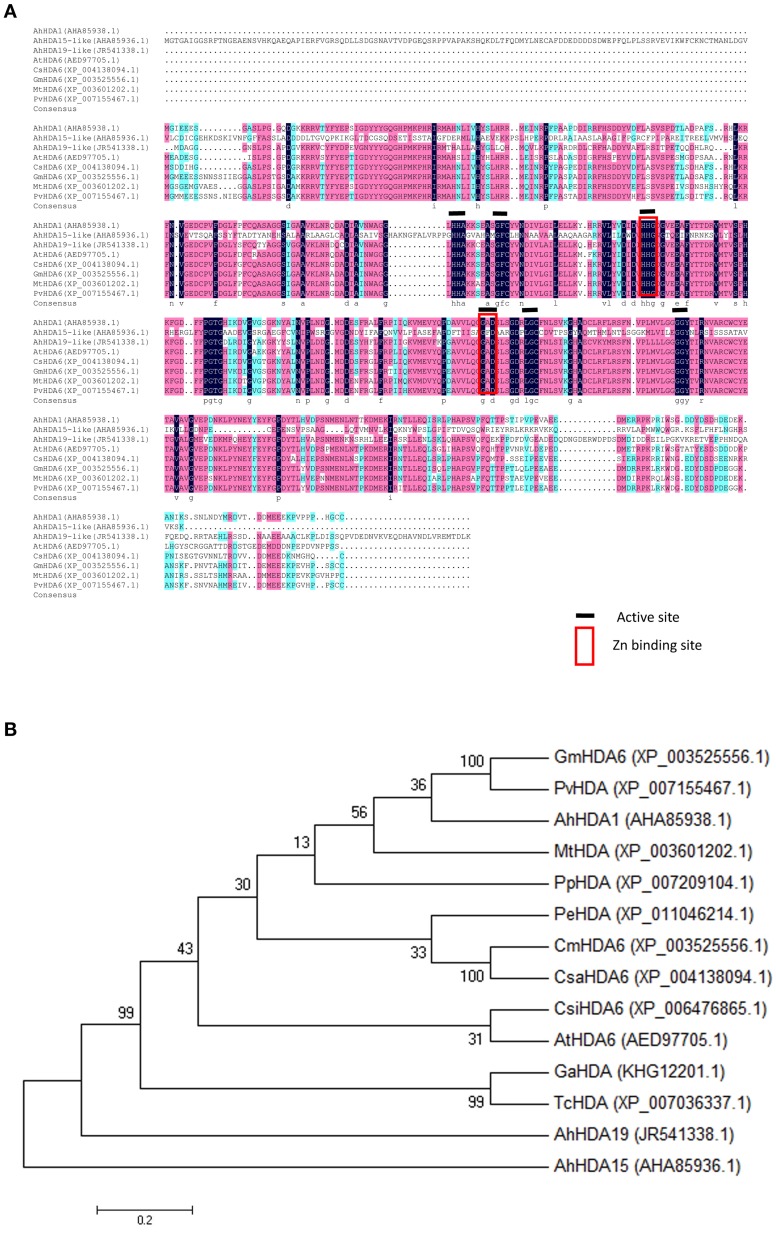
**Relatedness of peanut HDACs sequences to counterparts in other plants. (A)** Alignment of deduced amino acid sequence of peanut HDACs with other plant HDAC sequences. The degree of similarity of the amino acid residues at each residues at each aligned position is shaded black, red, blue, in decreasing order. GenBank accession numbers for each aligned HDAC protein are indicated in parenthesis. **(B)** Phylogenetic analysis of amino acid sequences of AhHDA1 and other plant HDACs. A multiple sequence alignment was performed using Clustal W and the phylogenetic tree was constructed via the Neighbor-Joining method in MEGA 4 software. Bootstrap values from 1000 replicates for each branch are shown. GenBank accession numbers: *Glycine max HDA6* (XP_003525556.1), *Phaseolus vulgaris HAD* (XP_007155467.1), *Arachis hypogaea HDA1* (JR541338.1), *Medicago truncatula HDA* (XP_003601202.1), *Prunus persica HDA* (XP_007209104.1), *Populus euphartica HDA* (XP_011046214.1). *Cucumis melo HDA6* (XP_00864523.1), *Cucumis sativas HDA6* (XP_004138094.1), *Citrus sinensis HDA6* (XP_006476865.1), *Arabidopsis thaliana HA6* (AED97705.1), *Gossypium arboretum HDA* (KHG12201.1), *Theobroma cacao HDA* (XP_007036337.1), *Arachis hypogaea HDA19-like* (AHA85936.1), *Arachis hypogaea HDA15-like* (AHA85936.1). The scale bar is 0.02.

Specific forward and reverse primers (ORF1-F and ORF1-R) were designed from *comp66763_c0* to isolate an *AhHDA1* cDNA as detailed in Materials and Methods. By sequence alignment, the predicted sequence of the AhHDA1 protein showed a high degree of similarity with other HDACs in the GenBank DNA database, and AhHDA1 possessed the same active site and Zn^2+^ binding sites as other plant HDACs (Figure 2A). AhHDA1 consists of a polypeptide of 467 amino acid residues with a calculated molecular weight of 52.37 kDa and an isoelectric point of 5.28. It can be deduced from Figure 2B that AhHDA1 is most similar to counterparts in eudicots, especially soybean.

The SWISS-MODEL tool was used to generate 3D structures for the AtHDA6 (encoded by the Arabidopsis *HDA6* gene) and AhHDA1 proteins (http://www.swissmodel.expasy.org; Figure S1). The 3D structures of both AhHDA1 and AtHDA6 were very similar, implying that the *AhHDA1* gene in peanut has a similar function to that of *AtHDA6* in Arabidopsis.

### Differential expression analysis in different tissues of *Arachis hypogaea* L.

**Figure 3:**
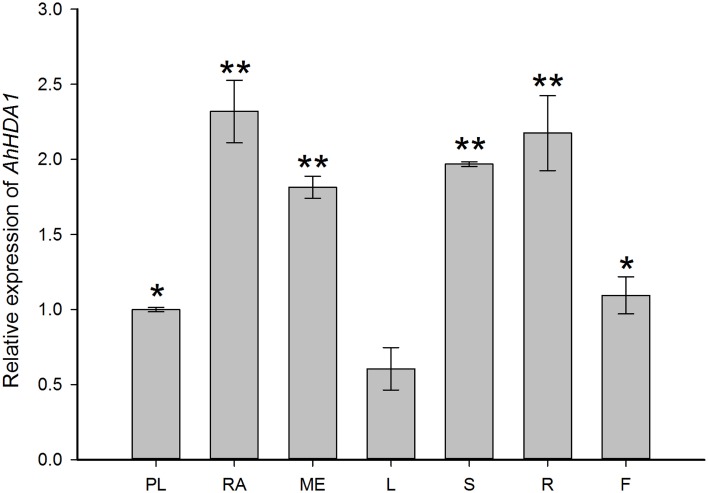
**Quantitative RT-PCR validations of** ***AhHDA1*** **expression in different peanut tissues**. Column chart showing expression of *AhHDA1* in PL, plumule; RA, radicle; ME, mesocotyl; L, leaf; S, stem; R, root; and F, flower, respectively. Plumules, radicles, and mesocotyls were taken from peanut embroys which had been cultivated for 7 days germination. Leaves stems and roots were taken from four-leaf stage peanut seedlings. Flowers were taken from peanuts during budding. All plants were grown as started in Materials and Methods. All values ± Standard Error (SE) for *n* = 3 biological replicates. Each graph displays the mean and SD of three independent experiments. ^*/**^, different from control as revealed by *t*-test, *p* < 0.05/*p* < 0.01.

Quantitative RT-PCR analysis was performed to examine the expression of *AhHDA1* in untreated embryos (plumule, radicle, and mesocotyl) and four-leaf seedlings (leaf, stem, root, and flower) (Figure 3). *AhHDA1* mRNA predominantly accumulates in radicle and mesocotyl of the embryo; similarly, in seedlings, *AhHDA1* mRNA predominantly accumulates in stem and root.

### Enhancement of *AhHDA1* transcript level in peanut leaves from four-leaf seedlings in response to osmotic stress, ABA, and histone deacetylase inhibitor

**Figure 4:**
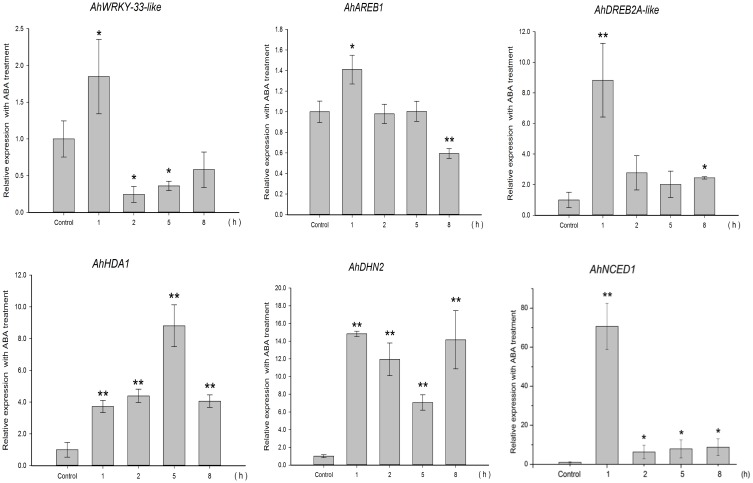
**Expression analyses of** ***AhHDA1*** **and stress resistance genes following ABA treatment by qRT-PCR**. Time points of 1, 2, 5, and 8 h were sampled to observe the changing trend. The untreated group was used as the control (no chemical treatment). Each graph shows the mean and SD of three independent experiments. ^*/**^, different from control as revealed by *t*-test, *p* < 0.0.5/*p* < 0.01.

**Figure 5:**
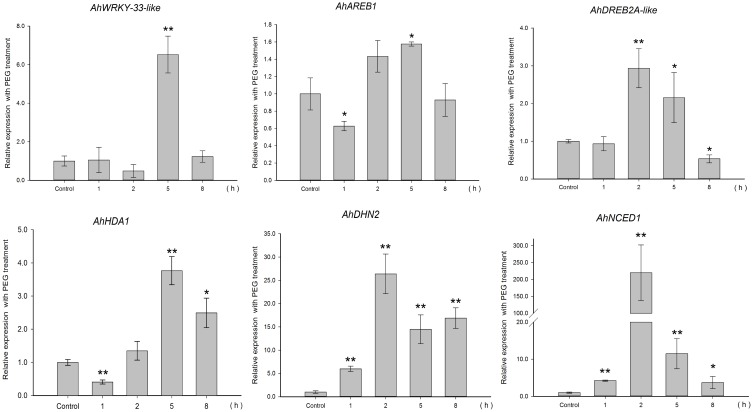
**Expression analyses of** ***AhHDA1*** **and stress resistance genes following PEG treatment by qRT-PCR**. Time points of 1, 2, 5, and 8 h were sampled to observe the changing trend. The untreated group was used as the control (no chemical treatment). Each graph shows the mean and SD of three independent experiments. ^*/**^, different from control as revealed by *t*-test, *p* < 0.0.5/*p* < 0.01.

**Figure 6:**
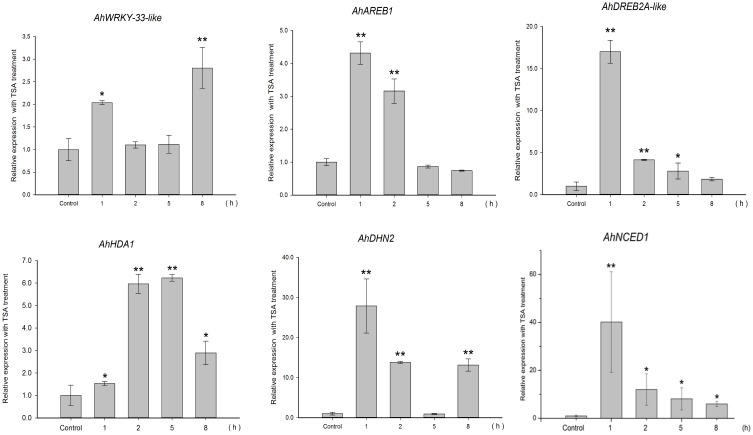
**Expression analyses of** ***AhHDA1*** **and stress resistance genes following TSA treatment by qRT-PCR**. Time points of 1, 2, 5, and 8 h were sampled to observe the changing trend. The untreated group was used as the control (no chemical treatment). Each graph shows the mean and SD of three independent experiments. ^*/**^, different from control as revealed by *t*-test, *p* < 0.0.5/*p* < 0.01.

To gain insight into the regulation of *AhHDA1*, qRT-PCR analyses were carried out in peanut leaves from four-leaf seedlings using gene-specific internal primers (Table 1). We investigated the changing trend of *AhHDA1* expression resulting from ABA treatment, as well as during the first rapid phase of water stress resulting from treatment with PEG. At the same time, the specific HDAC inhibitor TSA was used to examine the role of *AhHDA1* in the response to ABA and osmotic stress (Figure S4). Drought resistance genes were also analyzed with all these treatments. By comparison with the control group, we found that the expression of *AhHDA1* was enhanced by all three treatments (Figures 4**–6**). The *AhHDA1* transcript level increased to 4 times that of the control group at 1 h, and remained at a relatively high level from 2 to 8 h in ABA-treated plants. PEG and TSA treatments gave an expression profile almost identical to that of ABA-treated seedlings: *AhHDA1* expression in TSA groups increased from 1 h and stayed at a high level throughout the remainder of the experiment, while *AhHDA1* expression in PEG groups increased from 5 h, rather than 1 h, and stayed high at 8 h.

The expression profiles of TF genes (*AhAREB1, AhDREB2A*-like, *AhWRKY33*-like) and functional genes (*AhDHN2* and *AhNCED1)* were also determined in all three groups. It is clear from our results (Figures 4**–6**) that the expression patterns of these TF genes and functional genes in peanut leaves were very similar to that of *AhHDA1*, both of which show an initial increase followed by a decline in expression. More specifically, the expression of *AhAREB1, AhDREB2A*-like, and *AhWRKY33*-like began to increase at 1 h in both ABA and TSA groups and stayed at a high level or decreased from 2 h. *AhAREB1* and *AhWRKY33*-like expression began to increase from 5 h during PEG treatment, while the expression of *AhDREB2A*-like increased from 2 h. At the same time, the expression of *AhDHN2* and *AhNCED1*, the functional genes, began to increase from 1 h in all three treatment groups and maintained a high level of expression after 2 h.

### Histone deacetylase activity of recombinant AhHDA1 protein

**Figure 7:**
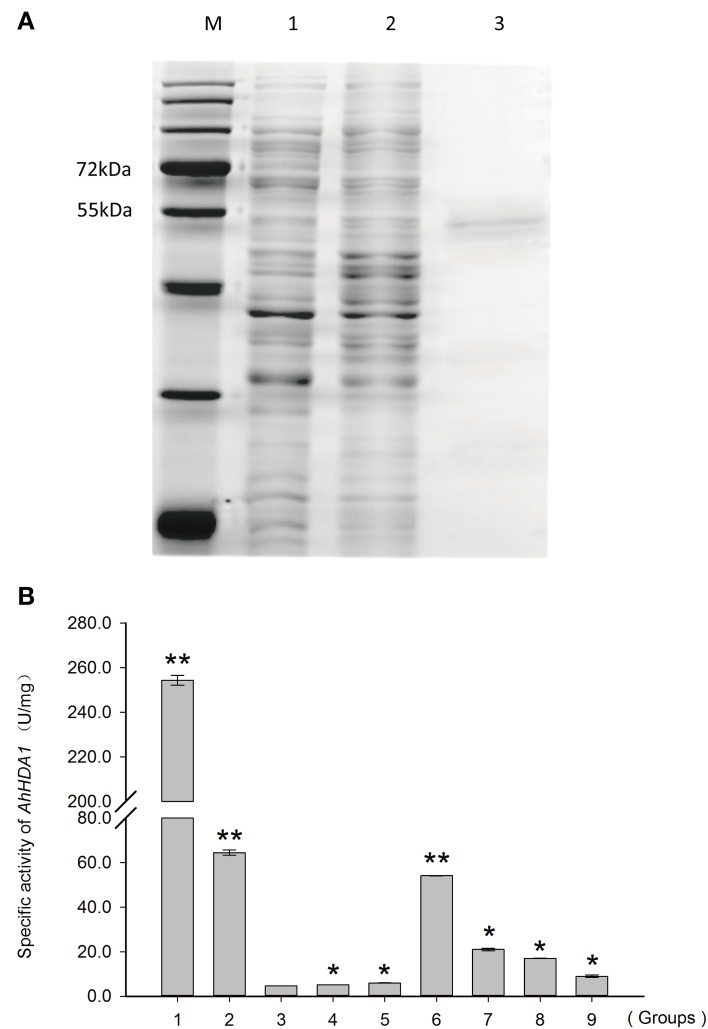
**HDAC activity of recombinant AhHDA1 produced in** ***E. coli*** **BL21. (A)** SDS-PAGE showing (1) total protein from *E. coli* cells expressing the recombinant plasmid pPROEX-AhHDA1 before induction by IPTG; (2) total protein from *E. coli* cells expressing recombinant plasmid pPROEX-AhHDA1 after induction by IPTG; for 20 h; and (3) purified AhHDA1 protein. **(B)** *In vitro* HDAC activity assay of the recombinant AhHDA1 protein. 1, positive control, extract from Hela cells; 2, extract from Hela cells treated with 4 μM TSA; 3, negative control, extract form *E. coli* cells containing plasmid pPROEX; 4, extract from *E. coli* cells containing plasmid pPROEX-AhHDA1 without IPTG; 5, extract from cells containing plasmid pPROEX after induction by IPTG; 6, extract from cells containing plasmid pPROEX-AhHDA1 after induction by IPTG for 20 h; 7, extract from cells containing plasmid pPROEX-AhHDA1 after induction by IPTG, but treated with 4 μM TSA for 20 h; 8, purified recombinant AhHDA1 protein; 9, purified recombinant AhHDA1 protein treated with 4 μM TSA. Each graph shows the mean and SD of three independent experiments. ^*/**^, different from control as revealed by *t*-test, *p* < 0.0.5/*p* < 0.01.

Recombinant AhHDA1 was produced in *E. coli* (Figure S2) as a polypeptide of about 53 kDa (**Figure 7A**). A total protein extract from *E. coli* expressing AhHDA1 was tested for HDAC activity (**Figure 7B**, Figure S3). The HDAC activity of an extract from cells containing plasmid pPROEX (i.e., the expression vector control) and the HDAC activity of an extract from cells containing plasmid pPROEX-AhHDA1 without IPTG treatment were 4.6 and 5.2 U/mg, respectively. When AhHDA1 expression was induced by IPTG treatment, HDAC activity of the cell extract increased to 54.1 U/mg; purified recombinant AhHDA1 protein gave an activity of 21.0 U/mg. When the HDAC inhibitor TSA was added to either the induced cell extract or to purified recombinant AhHDA1 protein, the HDAC activity decreased to 17.0 and 7.4 U/mg, respectively. The presence of HDAC activity correlated with expression of recombinant AhHDA1 protein after induction by IPTG, suggesting that the peanut protein is functional; the results also confirm that TSA effectively inhibits AhHDA1 activity.

## Discussion

### The consequences of osmotic stress and ABA treatment for histone acetylation of H3K9 and H3K14 and gene expression in *Arachis hypogaea* L.

Histone acetylation is a common modification of plant chromatin and plays a critical role in the epigenetic control of gene expression. It is involved in the response to both drought and ABA in various plants, including Arabidopsis, rice, and maize (Hu et al., 2011; Vlachonasios et al., 2011; Fang et al., 2014). Kim et al. have proposed that enrichment of H3K9ac, but not H3K14ac, correlates with gene activation in the coding regions of drought-responsive genes in Arabidopsis (Kim et al., 2008). However, in peanut, we found that water limitation resulted in increased acetylation of both H3K9 and H3K14, albeit at different time points. Thus, H3K9ac levels were significantly enhanced by 2 h of PEG treatment, and continued to increase throughout the experiment (up to 8 h). In contrast, H3K14ac levels increased, but not until 8 h after osmotic stress was imposed (Figure 1A). A different result was obtained with ABA: increased acetylation of H3K14 was observed after treatment for 5 h (Figure 1B), but there was relatively little effect on H3K9 acetylation for the duration of the experiment. Thus, the degree of acetylation in each case indicates that osmotic stress stimulates histone acetylation mainly at the H3K9 locus, whereas ABA induces histone acetylation primarily on H3K14. An explanation for this result might be that stress-responsive gene expression is governed by two different types of TF, AREB/ABFs, and DREB2A, which operate through the ABA-dependent and ABA-independent signaling pathways, respectively (Sreenivasulu et al., 2006; Fujita et al., 2011; Yoshida et al., 2014). Thus, the SnRK2-AREB (ABA-responsive element binding)/ABF pathway governs the majority of ABA-mediated AREB-dependent gene expression in response to osmotic stress during the vegetative stage of Arabidopsis (Fujita et al., 2011), while an ABA-independent but interactive pathway acts via the dehydration-responsive element binding (DREB) 2A TF (Sreenivasulu et al., 2006).

The effect of osmotic stress and exogenous ABA function on *AhHDA1* was examined and it was found to be up-regulated by both PEG and ABA early in the response to both treatments (Figures 4, 5). However, the mechanisms underlying these responses are not known, and therefore it is not clear whether ABA and osmotic stress act on *AhHDA1* via a common pathway or via independent pathways. Given that *AhHDA1* transcription begins to increase significantly after 1 h of ABA treatment, but not until 2 h after PEG treatment, and that H3K14ac increases from 1 h of ABA treatment, while H3K9ac increases from 2 h of PEG treatment, it is possible that ABA-dependent stress-responsive genes are activated through modification of the H3K14 locus, and ABA-independent stress-responsive genes are activated at the H3K9 locus. The increased *AhHDA1* expression induced by PEG or ABA might result from rapid changes in the HDAC and HAT “switches,” which re-balance histone and deacetylation.

### AhHDA1 is a RPD3/HDA1 histone deacetylase subfamily protein and is structurally similar to AtHDA6

Based on our understanding of the relationship between stress and histone acetylation, together with our analysis of RNA-seq data, we may surmise that HDAC activity in peanut plays an important role in the responses to both osmotic stress and ABA treatment. In this paper, we isolated and characterized *AhHDA1* from *A. hypogaea* L. cv Yueyou 7, a drought-resistant peanut variety we have reported on in previous studies (Fang et al., 2007). *AhHDA1* accumulates in the stem and root in seedlings, while it predominantly accumulates in the radicle and mesocotyl in the embryo (Figure 3). Both the predicted sequence and structure of the AhHDA1 protein appear to be well-conserved as judged by multiple sequence alignment, phylogenetic analysis and comparison of AtHDA6 and AhHDA1 ribbon diagrams (Figure 2, Figure S1). The HDAC activity of AhHDA1 was demonstrated by heterologous expression of recombinant protein in bacteria and this activity was inhibited by the specific HDAC inhibitor TSA. Using the Arabidopsis *HDA6* mutant *axe1-5* and HDA6 RNA-interfering plants, which display higher sensitivity to NaCl and ABA than wild type, Chen et al. found AtHDA6 to be involved in histone modifications that modulate seed germination and the salt stress response (Chen et al., 2010). *AtHDA6* mutations also result in transcriptional gene silencing, which influences the expression of auxin-inducible genes (Murfett et al., 2001). Given its structural relatedness to *AtHDA6, AhHDA1* may possess similar functions.

### The relationship between environmental stress, histone acetylation status and activity of stress resistance genes

TSA is an HDAC inhibitor that induces transient hyperacetylation of histones H2B, H4, and H3 (Waterborg, 2011). Drought-induced *RAB18, RD29B, HSP70* and four late embryogenesis abundant protein genes (*LEA*) are up-regulated by TSA in imbibing *A. thaliana* seeds (Tai et al., 2005). In our studies, TSA promoted the expression of *AhHDA1* (Figure 6) and induced acetylation of H3 (Figure S4). The expression patterns of three TF genes (*AhAREB1, AhDREB2A*-like, and *AhWRKY33*-like) and two functional genes (*AhDHN2* and *AhNCED1*) were also determined to study how TSA acted on stress resistance genes. TF gene expression was found to increase at an early time point after TSA treatment, then decreased as time went on. Although the expression profile of the functional genes was similar, the transcript level of these genes remained high relative to the control groups.

Because HDACs are inhibited by TSA which induces transient hyperacetylation of histone H3 (Figure 7, Figure S4) (Finnin et al., 1999), it seems reasonable to suppose that the up-regulated expression of *AhHDA1* following TSA treatment results from a feedback mechanism to re-establish the balance of histone acetylation and diacetylation in the plant. We might speculate that the mechanism of action of environmental stress, including osmotic stress and ABA signaling, on *AhHDA1* expression is as follows: histone acetylation is enhanced in peanut leaves soon after they are exposed to osmotic stress or ABA; subsequently, upstream TFs become activated and induce the expression of functional genes. Later, TF activity is modulated to a relatively insensitive state as the products of functional genes, such as the dehydrin AhDHN2, begin to protect plant cells from environmental stress damage.

Our work on *AhHDA1* has encompassed bioinformatic analysis of the gene, *in vitro* activity analysis of the corresponding recombinant protein and analysis of the effects of osmotic stress and ABA on *AhHDA1* expression. We conclude that *AhHDA1*, which is very similar to *AtHDA6*, is up-regulated by osmotic stress, ABA, and TSA. Future studies will focus on which genes undergo specific histone acetylation in response to water limitation and ABA treatment, and on an investigation of the critical genes in ABA-dependent and ABA-independent signaling pathways. These might help elucidate the molecular mechanisms of drought resistance, results that could be used to produce new varieties of crops for cultivation in water-limiting conditions.

## Author contributions

LCS, drafted the manuscript; BD, conducted the bioinformatics analysis; LL, LCS, BD, conceived and designed the experiments; LCS, BD, SL, LML, BH, YTZ, performed the experiments; BH, analyzed the data; LL, contributed reagents/materials/analysis tools.

## Funding

This work was supported by grants from the National Natural Science Foundation of China (No. 31471422 granted to LL).

### Conflict of interest statement

The authors declare that the research was conducted in the absence of any commercial or financial relationships that could be construed as a potential conflict of interest.
