# Supplementary material for: Isolation and characterization of an osmotic stress and ABA induced histone deacetylase in Arachis hygogaea
Source: Front Plant Sci. 2015 Jul 13;6:512. doi: 10.3389/fpls.2015.00512 (PMC4499716; doi:10.3389/fpls.2015.00512)
Supplement: Supplementary file 1 [file Table1.PDF]

Table S1. Primers for real-time quantitative PCR.

Gene Name	Primer Sequence (5'-3')	Size (bp)	GenBank Accession No.
AhAREB1	AhAREB1-F: ACAAGGGCAACCAGCATTAGG<br>AhAREB1-R: TCACCACCACCATACCAACCA	141	JF766571
AhDREB2A-like	AhDREB1-like-F: CAGCAGGCTTTGGCTTGGAACT<br>AhDREB1-like-R: GAAGAATGGTGGGAGAAACTGGAGAC	141	comp65625_c0
AhWRKY33-like	AhWRKY33-like-F: TGGACTTTTCGGCTACAC<br>AhWRKY33-like-R: CCTTCCTTTCATCCTTGG	146	comp69184_c3
AhNCED1	AhNCED1-F: TTACCTGTGGGATTGTTTGC<br>AhNCED1-R: ACATGAGCCTCTACTTCTGC	80	AJ574819
AhDHN2	AhDHN2-F: GTTACTTCCTTCCATTACGC<br>AhDHN2-R: GCTTATTCTCACCCTCCTTT	128	HM543578
AhHDA1	AhHDA1-F: GAGAGTGATGGGGATAGAAG<br>AhHDA1-R: GGAGTAGTGAACGATGAGGT	165	KC690279
Actin	Actin-F: GATTGGAATGGAAGCTGCTG<br>Actin-R: CGGTCAGCAATACCAGGGAA	134	KJ186104
ADH3	ADH3-F: GCTTCAAGAGCAGGTCACAAGT<br>ADH3-R: GAGACATCCTCCTTCGTGCATA	143	EG529529
