# Supplementary figures and images for: Isolation and characterization of an osmotic stress and ABA induced histone deacetylase in Arachis hygogaea
Source: Front Plant Sci. 2015 Jul 13;6:512. doi: 10.3389/fpls.2015.00512 (PMC4499716; doi:10.3389/fpls.2015.00512)

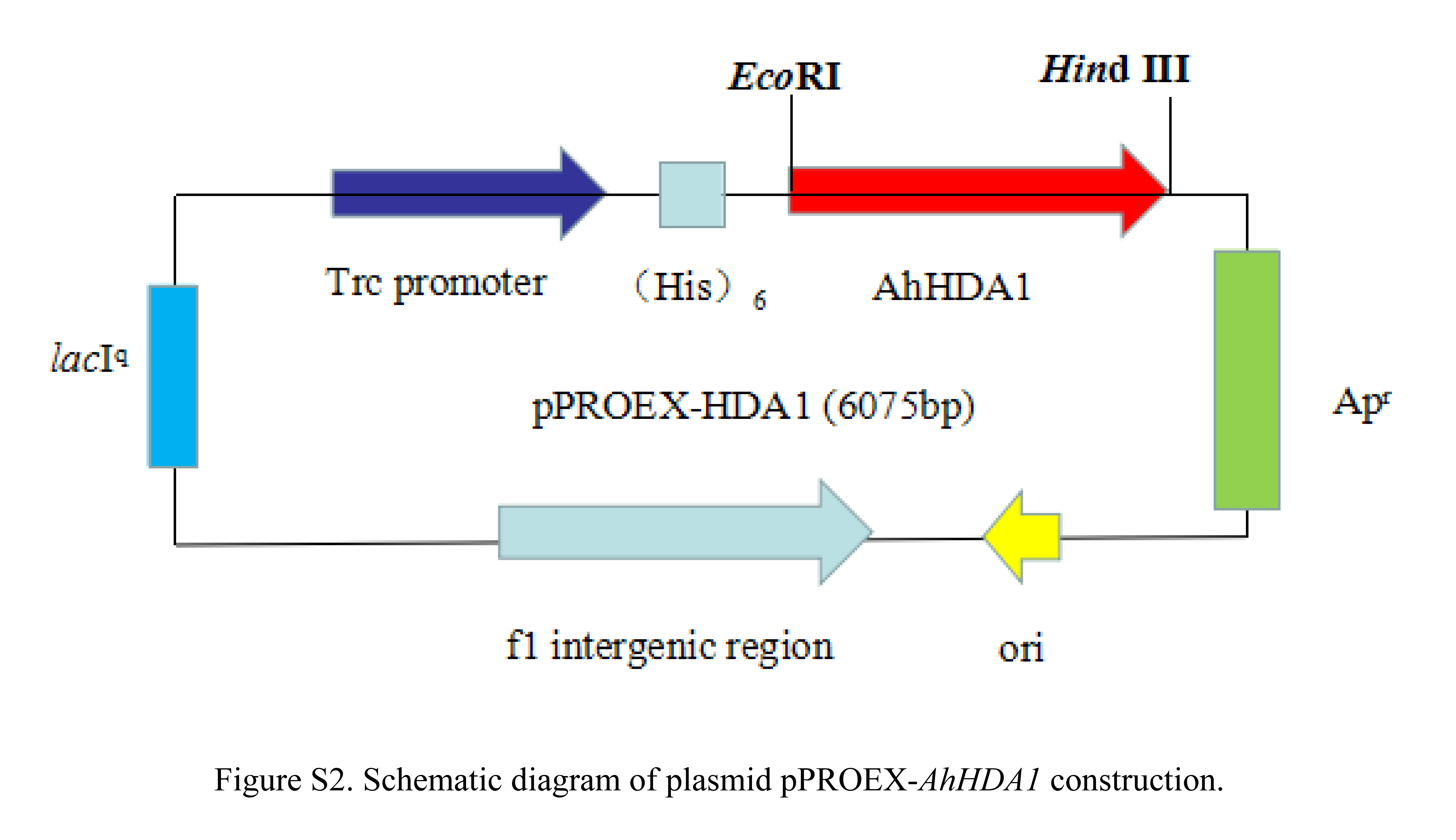

Supplement: Supplementary file 3 [file Image2.TIF]

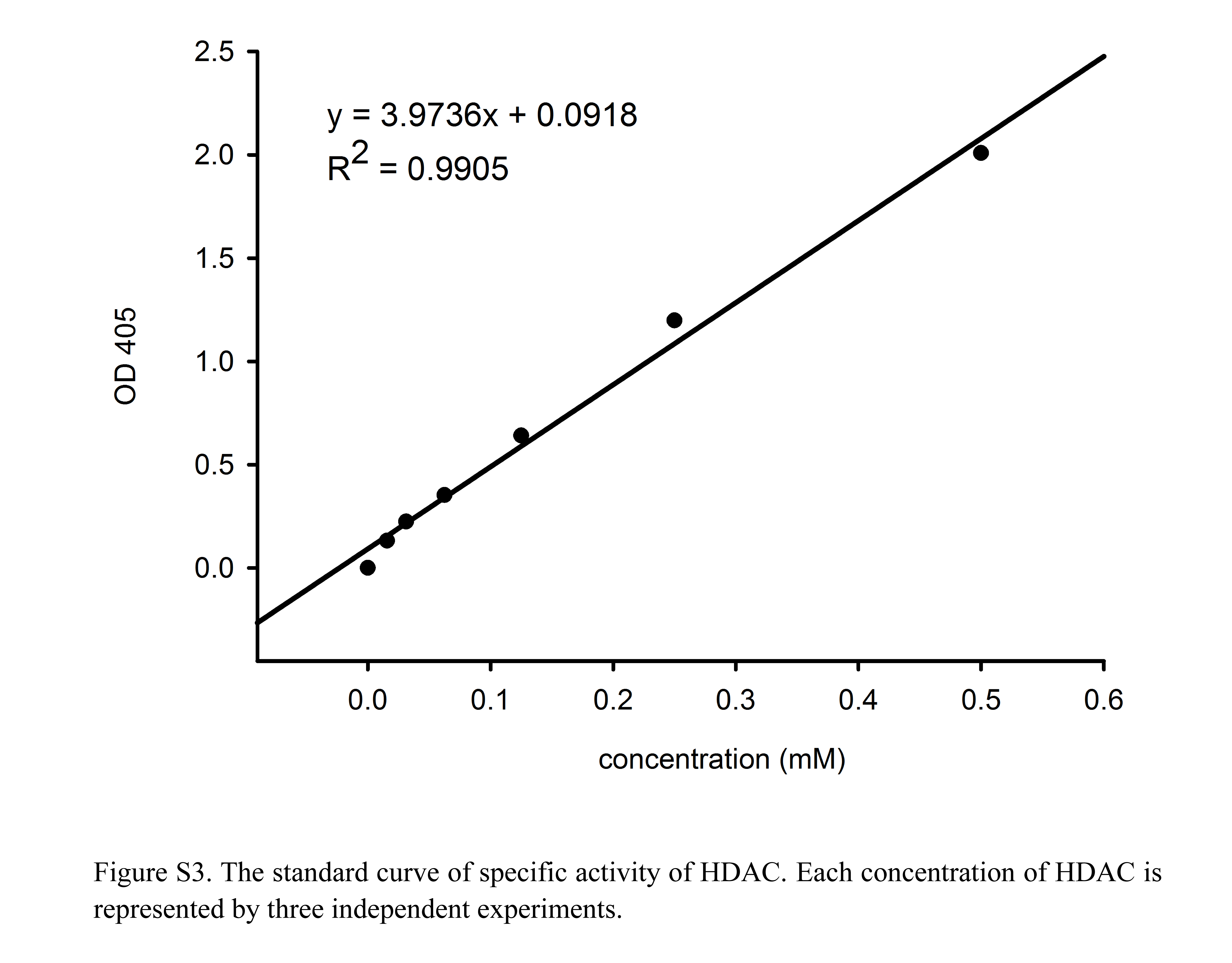

Supplement: Supplementary file 4 [file Image3.TIF]

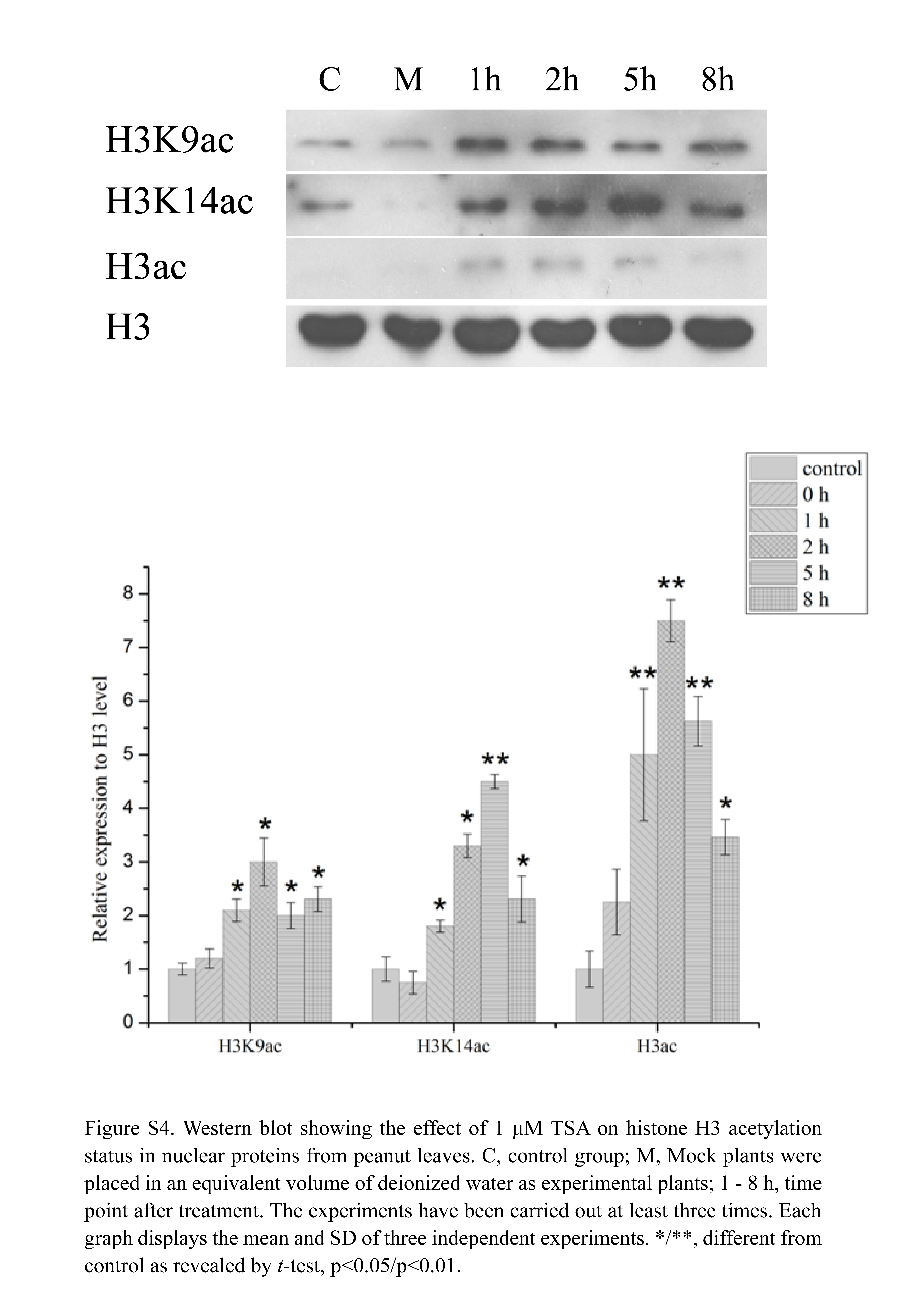

Supplement: Supplementary file 5 [file Image4.TIF]
